# NLRC4 regulates caspase-1 and IL-1beta production in a CD11b^low^Ly6G^low^ population of cells required for resistance to *Pseudomonas aeruginosa* keratitis

**DOI:** 10.1371/journal.pone.0185718

**Published:** 2017-09-29

**Authors:** Sharon A. McClellan, Andrew Jerome, Susmit Suvas, Linda D. Hazlett

**Affiliations:** 1 Department of Anatomy and Cell Biology, Wayne State University School of Medicine, Detroit, MI, United States of America; 2 Department of Ophthalmology, Wayne State University School of Medicine, Detroit, MI, United States of America; Virginia Polytechnic Institute and State University, UNITED STATES

## Abstract

*Psbetaeudomonas (P*.*) aeruginosa* infection of the cornea in BALB/c mice does not result in perforation and the mice have been classified as resistant. However, regulation of this response via inflammasome activation remained untested. Therefore, BALB/c mice were infected with *P*. *aeruginosa* ATCC strain 19660 and NLRP3 and NLRC4 protein tested by ELISA. Since NLRC4 vs NLRP3 protein levels were significantly higher in the corneas of BALB/c at 1 and 5 days postinfection we used silencing to knockdown NLRC4. Silencing NLRC4 vs scrambled siRNA treatment exacerbated disease in BALB/c mice, reduced myeloperoxidase levels and elevated bacterial plate counts at 5 days postinfection. It also increased pro IL-1beta, but reduced total protein for IL-1beta and IL-18 at 5 days postinfection. Flow cytometry to identify cells affected by silencing, showed reduced caspase-1 levels in a CD11b^low^Ly6G^low^ population of cells, (but not PMN or macrophages) from the infected cornea of siNLRC4 treated mice that produced less mature IL-1beta. These data provide evidence that the NLRC4 inflammasome contributes to resistance through regulation of caspase-1, IL-1beta and IL-18 in a CD11b^low^Ly6G^low^ population of cells.

## Introduction

*Pseudomonas aeruginosa* (*P*. *aeruginosa*), an opportunistic Gram-negative pathogen, is one of the most common causes of microbial keratitis in extended-wear contact lens users and those who are immunocompromised [1]. Animal models of bacterial keratitis, including use of a murine model of susceptible (cornea perforates) C57BL/6 (B6) and resistant (cornea heals) BALB/c mice have provided information on ocular immune defenses against *P*. *aeruginosa* [2, 3]. In this regard, IL-1beta plays an important role in the induction of the immune response and is upregulated postinfection (p.i.) in the cornea of both groups of mice [4]. In a classical response to proinflammatory stimuli, the IL-1 beta precursor is induced in monocytes, macrophages and polymorphonuclear neutrophils (PMN) and processed into its biologically active form by caspase-1. The protease, caspase-1 is expressed as an inactive zymogen that is activated when cleaved by multi-protein complexes known as inflammasomes [5].

Inflammasomes are a functional subgroup of the Nod-like receptor (NLR) family and are considered “danger” sensor complexes that trigger immune system activation [6]. Inflammasome activation involves the oligomerization of an NLR scaffold, followed by the recruitment, clustering, and autoactivation of a proinflammatory caspase, caspase-1, usually via a protein adaptor ASC [7]. Upon activation, caspase-1 mediates the maturation and secretion of proinflammatory cytokines such as IL-1beta and IL-18. These caspase-1 functions coordinate host-protective inflammatory and antimicrobial responses. IL-1beta and IL-18 drive proinflammatory responses, including the recruitment and activation of phagocytes [8]. The most studied of the inflammasomes are NLR family pyrin domain containing 3 (NLRP3) and NLR family CARD domain containing protein 4 (NLRC4). Specific Gram-negative bacteria encoding type 3 or 4 secretion systems, such as *P*. *aeruginosa*, trigger activation of the NLRC4 inflammasome [9, 10] while ATP, bacterial toxins and particulate matter activate the NLRP3 inflammasome [11, 12]. The role of NLRC4 in disease has been shown to be both causative and protective. A missense mutation in NLRC4 has been implicated in autoinflammatory syndromes that cause sterile inflammation in the absence of any signs of autoimmune responses. NLRC4 has been shown by others to be protective in models of respiratory melioidosis [13] and *Salmonella* infection [14].

Despite these studies, the role of the inflammasome in the resistance response of BALB/c mice to *P*. *aeruginosa* corneal infection remains untested. In that regard, our data provide evidence that NLRC4 is upregulated significantly in BALB/c mice after corneal infection with *P*. *aeruginosa*. Knockdown of NLRC4 resulted in worsened corneal disease, a decreased PMN infiltrate and increased viable bacterial plate count in the cornea of these mice. Silencing NLRC4 also lead to elevated levels of pro IL-beta and reduced levels of total IL-1beta and IL-18 protein. Flow cytometry analysis revealed a population of cells (CD11b^low^Ly6G^low^) that were neither PMN nor macrophages. When compared with macrophages and PMN, CD11b^low^Ly6G^low^ cells had a significant reduction in caspase-1 and NLRC4 expression and produced less mature IL-1betaafter silencing NLRC4. They also had a minimal expression of CD3 and NK1.1 molecules, and were less granulocytic (lower side scatter) than a CD45^high^ population of cells in the infected corneas. Together, these results provide evidence that CD11b^low^Ly6G^low^ cells have an immature myeloid cell phenotype and that the NLRC4 inflammasome in these cells contributes to the resistance response of BALB/c mice.

## Materials and methods

### Animals and experimental infection model

Eight-week-old female BALB/c mice (purchased from the Jackson Laboratory, Bar Harbor, ME) were housed in accordance with the National Institutes of Health guidelines. Animals were treated humanely and in compliance with the Association for Research in Vision and Ophthalmology (ARVO) Statement for the Use of Animals in Ophthalmic and Vision Research. The protocol was approved (with justification for the use of ether) by the Institutional Animal Care and Use Committee (IACUC) of Wayne State University (Protocol #A 07-07-15). All procedures were performed under ether anesthesia alone (as it has proved optimum to allow corneal wounding and application of the bacterial inoculum, with a quick recovery time of about 30 sec/mouse), and all efforts were made to minimize pain.

*P*. *aeruginosa* strain 19660 (American Type Culture Collection, Manassas, VA) was grown in peptone tryptic soy broth (PTSB) medium in a rotary shaker water bath at 37°C, 150 rpm for 18h to an optical density (measured at 540 nm) between 1.3–1.8. Bacterial cultures were pelleted by centrifugation at 5,500xg for 10 minutes. Pellets were washed once with sterile saline, centrifuged, resuspended and diluted in sterile saline to a final concentration of 1 X 10^6^ CFU/microliter [15]. For each experiment, animal sample size was determined from previous experience with similar studies, allowing statistically relevant comparisons. Mice were anesthetized using ethyl ether, placed beneath a stereoscopic microscope at x40 magnification and the left cornea scarified by making three 1-mm incisions with a sterile 25^5/8^-gauge needle. A 5 microliteraliquot of the bacterial suspension was applied topically to initiate infection. At the end of the experiment, mice were euthanized by cervical dislocation followed by pneumothorax, approved by the IACUC at Wayne State University.

### Ocular response to bacterial infection

An established scale [16] was used to grade disease and a clinical score assigned at 1, 3 and 5 days p.i. Clinical scores were used to statistically compare the disease severity at each time point and were designated as: 0 = clear or slight opacity, partially or fully covering the pupil; +1 = slight opacity, fully covering the anterior segment; +2 = dense opacity, partially or fully covering the pupil; +3 = dense opacity, covering the entire anterior segment; and +4 = corneal perforation or phthisis. Photogramicphs using a slit lamp camera were taken at 5 days p.i. to confirm and document the disease response.

### Knock down of NLRC4

Use of small interfering RNA (siRNA) in vivo has been described [17] and was used for this study as currently no knockout mouse is commercially or privately available. In these studies, siRNA targeting NLRC4 or siRNA for a non-targeting scrambled sequence (negative control) (Santa Cruz Biotechnology, Santa Cruz, CA) was injected subconjunctivally (5 microliter/mouse, 8 micromolar concentration) into the left eye of BALB/c mice on the day before infection, then applied topically onto infected corneas (5 microliter/mouse, 4 micromolar) once on the day of infection and twice on 1 day p.i. The efficacy and specificity of silencing NLRC4 was tested by ELISA. The siRNAs used in this study were shorter than 21 nucleotides in length to avoid nonspecific siRNA suppression effects via cell surface TLR3 [18].

### Enzyme-linked immunosorbent assay (ELISA)

BALB/c mice (n = 5 mice/group/time) were sacrificed on 1 and 5 days p.i. and the normal and infected corneas harvested. Individual corneas were homogenized in 500 microliter PBS with 0.1% Tween 20 with a protease inhibitor cocktail (Roche Diagnostics, Indianapolis, IN) and centrifuged at 12,000x*g* for five minutes. A 100 microliter aliquot of each supernatant was assayed in duplicate by ELISA to quantify NLRP3 (Antibodies on-line, Atlanta, GA) and NLRC4 (My BioSource, San Diego, CA) protein. siNRLC4 and scrambled siRNA treated BALB/c mice (n = 5 mice/group/time) were sacrificed on 1 and 5 days p.i. and the normal and infected corneas harvested processed and stored as described above and assayed for NLRC4, IL-1beta and IL-18 (MBL, Woburn, MA) protein. The pro form of IL-1beta was detected using a then available kit specifically designed to detect the uncleaved form of IL-1beta (Affymetrix eBioscience, San Diego, CA), a separate kit designed to detect both cleaved and uncleaved forms was used to detect total IL-1beta (R&D Systems, Minneapolis, MN). Supernatants and cell lysates from cells sorted and collected following flow cytometric analysis, described below, were assayed for NLRC4 and total IL-1beta. All ELISA kits were run following the manufacturer’s instructions. Sensitivities of the assays were as follows: 1.56 pg/ml (NLRP3), 50 pg/ml (NLRC4), 25 pg/ml (pro-IL-1beta), 2.31 pg/ml (total IL-1beta), and 25 pg/ml (IL-18).

### Western blotting

Western blotting was performed as described previously [19, 20]. Briefly, following siRNA treatment at 5 days p.i, infected and non-infected corneas were harvested. A corneal lysate was made in PBS containing a protease and phosphatase inhibitor cocktail (Thermoscientific, Rockford, IL) by sonication followed by centrifugation at 12,000x*g* for 20 min. The total protein concentrations of the corneal lysates were determined using a Micro BCA™ protein assay kit (Thermoscientific). Total protein samples (50 micrograms) were resolved on 16% SDS-PAGE in Tris-glycine-SDS buffer (25 mM Tris, 250 mM glycine, and 0.1% SDS) and electro-blotted onto a 0.2 micrometers nitrocellulose membrane (Millipore, Billerica, MA). After blocking for 1h in 5% MTBST (TBS containing 0.05% Tween 20 and 5% nonfat milk), the blots were probed with primary antibodies for IL-1beta (Santa Cruz Biotechnology) and IL-18 (Rockland, Limerick, PA) (1:1000) overnight at 4°C. The membranes were washed three times with TBST (TBS containing 0.05% Tween 20) and incubated with horseradish peroxidase conjugated secondary antibodies (Cell Signaling, Danvers, MA) diluted in 5% MTBST at room temperature for 1h. Protein bands were visualized with Supersignal West Femto Chemiluminescent Substrate (Thermoscientific).

### Quantitation of PMN in cornea

An assay for myeloperoxidase (MPO) was used to quantitate PMN number in the cornea of siNRLC4 and scrambled siRNA treated BALB/c mice. Individual corneas were removed at 1 and 5 days p.i. and homogenized in 1.0 ml of 50 mM phosphate buffer (pH 6.0) containing 0.5% hexadecyltrimethyl-ammonium (Sigma, St Louis, MO). Samples were freeze-thawed four times and after centrifugation, 100 microliters of the supernatant was added to 2.9 ml of 50 mM phosphate buffer containing *o*-dianisidine dihydrochloride (16.7 mg/ml, Sigma) and hydrogen peroxide (0.0005%). The change in absorbance was monitored at 460 nm for 5 min at 30 sec intervals. The slope of the line was determined for each sample and units of MPO/cornea calculated. One unit of MPO activity is equivalent to ~2x10^5^ PMN [21].

### Bacterial plate count

Mice were sacrificed on 1 and 5 days p.i., and infected corneas of siNLRC4 and scrambled siRNA treated BALB/c mice (n = 5/group/time) were harvested. Each cornea was homogenized in 1 ml sterile saline containing 0.25% BSA. A 100 microlitersof the corneal homogenate was serially diluted 1:10 in sterile saline containing 0.25% BSA. Selected dilutions were plated in triplicate on Pseudomonas isolation agar plates (Becton-Dickinson, Franklin Lakes, NJ). Plates were incubated overnight at 37°C and the number of viable bacteria manually counted. Results are reported as log_10_ CFU/cornea ± SEM.

### Flow cytometric analyses

Individual corneas from siNLRC4 and scrambled siRNA control treated mice (n = 5/group) were harvested on day 5 p.i. Individual corneas were kept in sterile tubes containing 250 microlitersRPMI 1640 medium without serum. Liberase TL (Roche, Indianapolis, IN, 2.5 mg/ml) was added to each tube followed by incubation at 37°C for 45 min on a Disruptor Genie. At the end of the incubation period, samples were triturated using a 3 ml syringe plunger, and passed through a 70 micrometer cell strainer. Finally, the single-cell suspension was washed with 5 ml RPMI 1640 containing 10% FBS and was pelleted at 315 ×g for 8 min in a refrigerated centrifuge. Cells were stained for cell surface CD45, CD11b, Ly6G, Ly6C, F4/80, CD3, and NK1.1 molecules. The following antibodies were used for cell surface staining: PerCP-Cy5.5–conjugated rat anti-mouse CD11b (M1/70,1:200), PE-Cy7-conjugated rat anti-mouse CD45 (30-F1, 1:200), APC-conjugated rat anti-mouse macrophage (F4/80, 1:200), PE-conjugated rat anti-mouse Ly6G (1A8, 1:400), APC-conjugated hamster anti- mouse CD3 (145-2C11, 1:200), and PE-conjugated mouse anti-mouse NK1.1 (PK136, 1:200). All antibodies were purchased from BD Biosciences (San Jose, CA). Samples were acquired on LSRFortessa flow cytometer (Beckton Dickinson, San Jose, CA). Data were analyzed using FlowJo software.

### Detection of activated caspase-1

Caspase-1 activation was detected in the subsets of CD45 expressing immune cells using a FAM-FLICA^TM^ Caspase-1 assay kit per the manufacturer’s instructions (Immunochemistry Technologies, Bloomington, MN). Briefly, single cell suspensions of infected corneas from siNLRC4 and scrambled siRNA control treated groups of mice were incubated with FAM-FLICA caspase-1 at 37°C, 5% CO_2_ for 45 min. At the end of the incubation period, cells were washed twice with 1x wash buffer provided in the kit followed by blocking of the Fc receptors with anti-mouse CD16/32 antibody on ice. Cell surface staining for CD45, CD11b, Ly6G, and F4/80 molecules was carried out and after, samples were immediately acquired using a LSRFortessa flow cytometer. Data were analyzed using FlowJo software and presented as mean fluorescence intensity (MFI) ± SEM.

### Sorting cells from the infected cornea

The cells of interest in infected corneas from siNLRC4 and scrambled control groups of mice were sorted on day 5 p.i. using a Sony SY32900 cell sorter (San Jose, CA). The gating strategy for Ly6G in FACS plots was applied on the basis of isotype antibody staining, as shown in S1 Fig and the level of expression of Ly6G. Briefly, single cell suspensions of the infected corneas from both groups were stained for the cell surface molecules as described above followed by sorting of macrophages, PMN and CD11b^low^Ly6G^low^ cell populations and collection in complete RPMI medium. Without further stimulation, equal numbers of sorted cells were plated and cultured at 37°C, 5% CO_2_ for 18h. At the end of the incubation period the contents of each well was removed and transferred to a microcentrifuge tube. The cells were pelleted by centrifugation, the supernatant transferred to another tube and the cell pellets lysed. The culture supernatant and cell lysates from each cell type were assayed by ELISA for NLRC4 and total IL-1beta.

### Statistical analysis

The difference in clinical score between two groups at each time was tested using the Mann-Whitney U test. An unpaired, two-tailed Student’s t-test was used to determine the statistical significance of the ELISA, MPO, bacterial plate count and flow data and considered significant at *p*≤0.05. All experiments were repeated at least once to ensure reproducibility and data from a representative experiment are shown as mean ± standard error of the mean.

## Results

### Inflammasome proteins and silencing of NLRC4 in BALB/c mice

**Fig 1:**
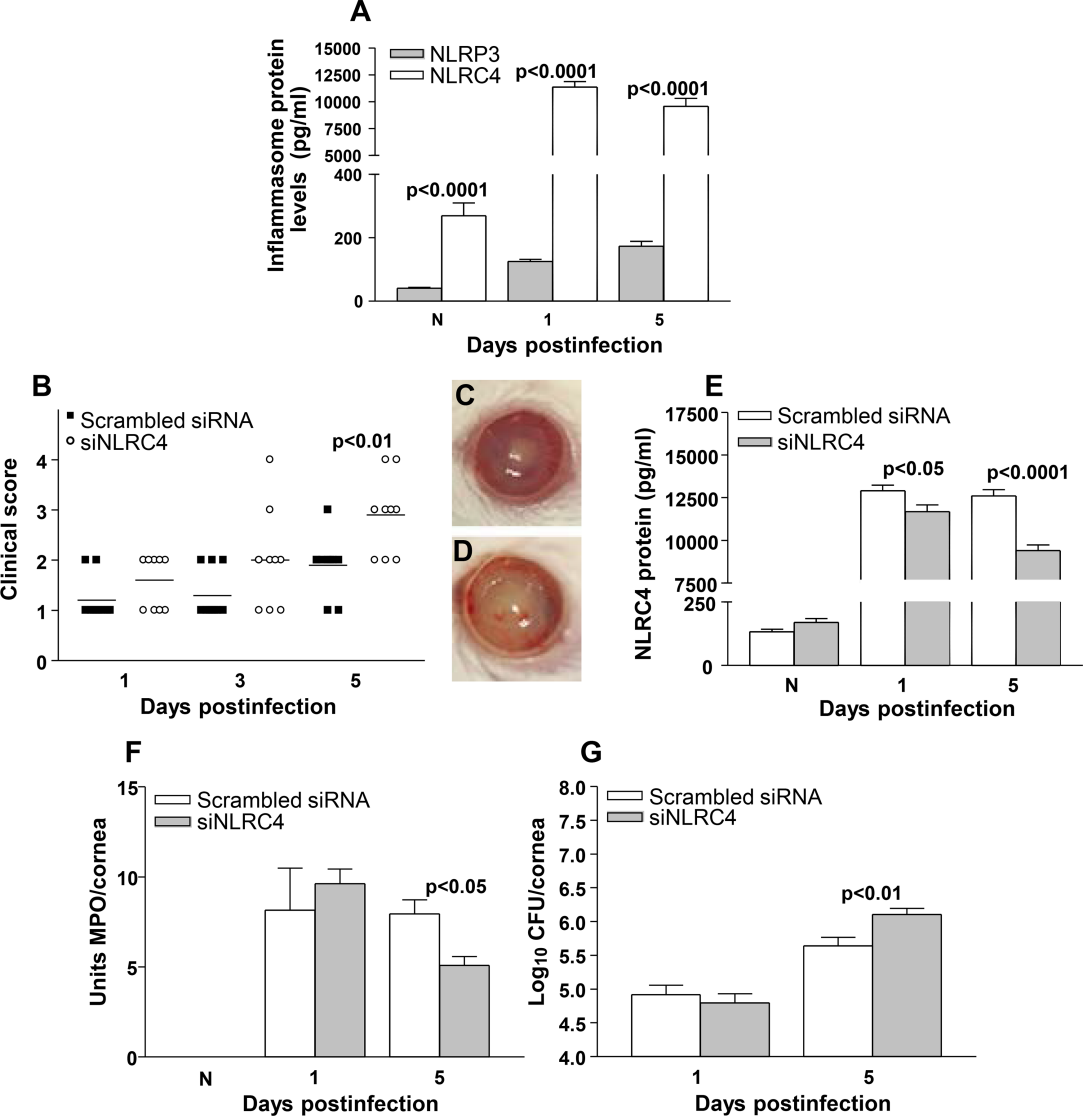
Expression of inflammasome proteins and silencing of NLRC4 in BALB/c mouse cornea. (A) ELISA analysis showed significantly more NLRC4 protein in the normal uninfected mouse cornea and at 1 and 5 days p.i. when compared to NLRP3 protein levels (n = 5/group/time). (B) Clinical score showed that treatment with siRNA targeting NLRC4 resulted in significantly more disease at 5 days p.i. with no difference at 1 and 3 days p.i. (n = 10/group/time). Data were analyzed using a non-parametric Mann-Whitney *U* test. Horizontal lines represent mean values. Photographs taken with a slit lamp camera of representative corneas at 5 days p.i. show less disease in (C) scrambled siRNA vs (D) siNLRC4 treated mice. (E) ELISA analysis confirmed inhibition of NLRC4 protein showing significantly reduced levels in corneas at 1 and 5 days p.i. in siNLRC4 vs scrambled siRNA treated mice (n = 5/group/time). No difference was detected in normal, uninfected corneas between treatment groups. (F) MPO assay detected significantly fewer PMN in the corneas of siNLRC4 treated compared with scrambled control treated mice at 5 days p.i. with no difference detected in normal, uninfected corneas or at 1 day p.i. (n = 5/group/time). (G) Bacterial plate counts showed significantly more viable bacteria in the corneas of siNLRC4 treated compared with scrambled control treated mice at 5 days p.i. but no difference was found at 1 day p.i. between groups (n = 5/group/time). Data (E-G) are mean ± SEM and were analyzed using a two-tailed Student’s *t* test.

ELISA assays were used to determine NLRP3 and NLRC4 protein expression levels in the normal uninfected cornea and at 1 and 5 days p.i. (Fig 1A) of BALB/c mice. NLRC4 vs NLRP3 protein levels were significantly higher (p<0.0001) in the normal cornea and in the infected cornea at 1 and 5 days p.i. The role of NLRC4 was tested using siRNA treatment to knock down its expression. Clinical scores were significantly higher in the cornea of siNLRC4 treated mice when compared to scrambled siRNA treated controls at 5 days p.i. (p<0.01). No significant difference in disease severity was seen between groups at 1 or 3 days p.i. (Fig 1B). Representative photographs taken with a slit lamp camera at 5 days p.i. of the corneas of scrambled siRNA (+2, Fig 1C) and siNLRC4 (+3, Fig 1D) treated mice are shown to illustrate disease severity. Efficacy and specificity of the siRNA treatment was confirmed by ELISA analysis. Protein levels of NLRC4 in the cornea were significantly decreased in siNLRC4 treated mice at 1 and 5 days p.i. when compared to the scrambled siRNA treated mice (p<0.05 and 0.0001, respectively, Fig 1E). No difference was detected between groups in normal, uninfected corneas. The number of PMN infiltrating the cornea after infection was quantitated by MPO assay. Significantly less MPO activity was detected in the corneas of siNLRC4 compared to scrambled siRNA control treated mice at 5 days p.i. (p<0.05, Fig 1F) with no difference between groups at 1 day p.i. No MPO activity was detected in normal, uninfected corneas. Viable bacterial plate counts also were done (Fig 1G) and more viable bacteria (~0.5 log) were found in the corneas of the siNLRC4 treated mice at 5 days p.i. when compared to scrambled siRNA treated controls (p<0.01). No difference in bacterial load was detected between groups at 1 day p.i.

### ELISA and Western blot assays for IL-1beta and IL-18

**Fig 2:**
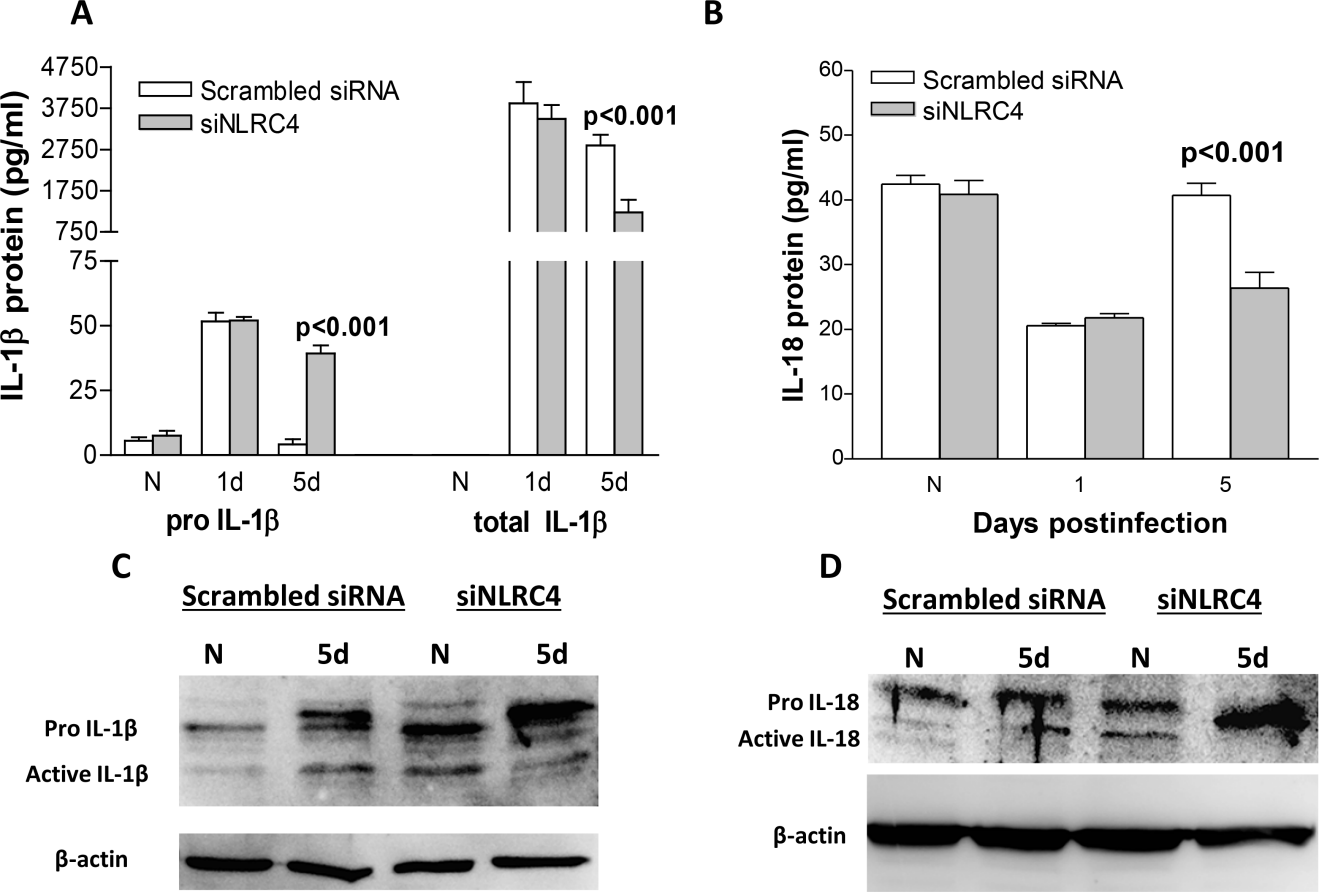
ELISA and Western blot assays for IL-1beta and IL-18. (A) Protein levels of pro IL-1beta (using a specific kit for analysis as described in Methods) were significantly elevated at 5 days p.i. after silencing NLRC4 compared with scrambled control treatment, with no difference seen between the groups at 1 day p.i. or for normal, uninfected corneas. Total IL-1beta protein levels were significantly reduced at 5 days p.i. after silencing NLRC4 compared with scrambled control treatment, with no difference seen between the groups at 1 day p.i. or for normal, uninfected corneas (n = 5/group/time). Total IL-18 protein levels (B) were significantly reduced at 5 days p.i. after silencing NLRC4 compared with scrambled control treatment, with no difference seen between the groups at 1 day p.i. or for normal, uninfected corneas (n = 5/group/time). Data are mean ± SEM and were analyzed using a two-tailed Student’s *t* test. Western blot data for IL-1beta(C) and IL-18 (D) at 5 days p.i., showed increased levels of pro-forms for both cytokines and reduction in their mature (secreted) forms only in the siRNA NLRC4 treated samples, fully supporting the ELISA analyses.

Since silencing NLRC4 resulted in worsened disease, reduced PMN infiltration (MPO assay) and elevated bacterial load in the cornea, we next tested expression of cytokines IL-1beta and IL-18, known to be upregulated by inflammasome activation (Fig 2A and 2B). Levels of pro-IL-1beta, the uncleaved, immature form of secreted (mature) IL-1beta levels in cornea did not differ between silenced and scrambled siRNA treated mice at 1 day p.i. nor in the normal, uninfected cornea. Significantly more pro-IL-1beta was detected in the cornea of the siNLRC4 compared to scrambled siRNA treated mice at 5 days p.i. (p<0.001, Fig 2A). However, total IL-1beta, a combination of both pro- and mature forms of the cytokine, was significantly reduced at 5 days p.i. (p<0.001) in the corneas of the NLRC4 silenced vs scrambled control treated mice. No significant difference was seen between groups at 1 day p.i, and no IL-1beta was detectable in normal, uninfected corneas as it has been shown previously that IL-1beta is not constitutively expressed in the cornea but upregulated by infection with the bacterium [4]. After silencing, levels of IL-18 protein (Fig 2B) also were significantly reduced at 5 days p.i. in the cornea of siNLRC4 treated compared to the cornea of scrambled control treated mice (p<0.001), while no difference was detected in the normal, uninfected cornea or at 1 day p.i. between groups. Western blot was used and confirmed the ELISA data for both IL-1beta(Fig 2C) and IL-18 (Fig 2D). These data indicate that at 5 days p.i. less pro-IL-1beta is being cleaved to the mature form and less mature IL-18 is being secreted after NLRC4 silencing compared to all other groups.

### Flow cytometry analysis for caspase-1 expression

**Fig 3:**
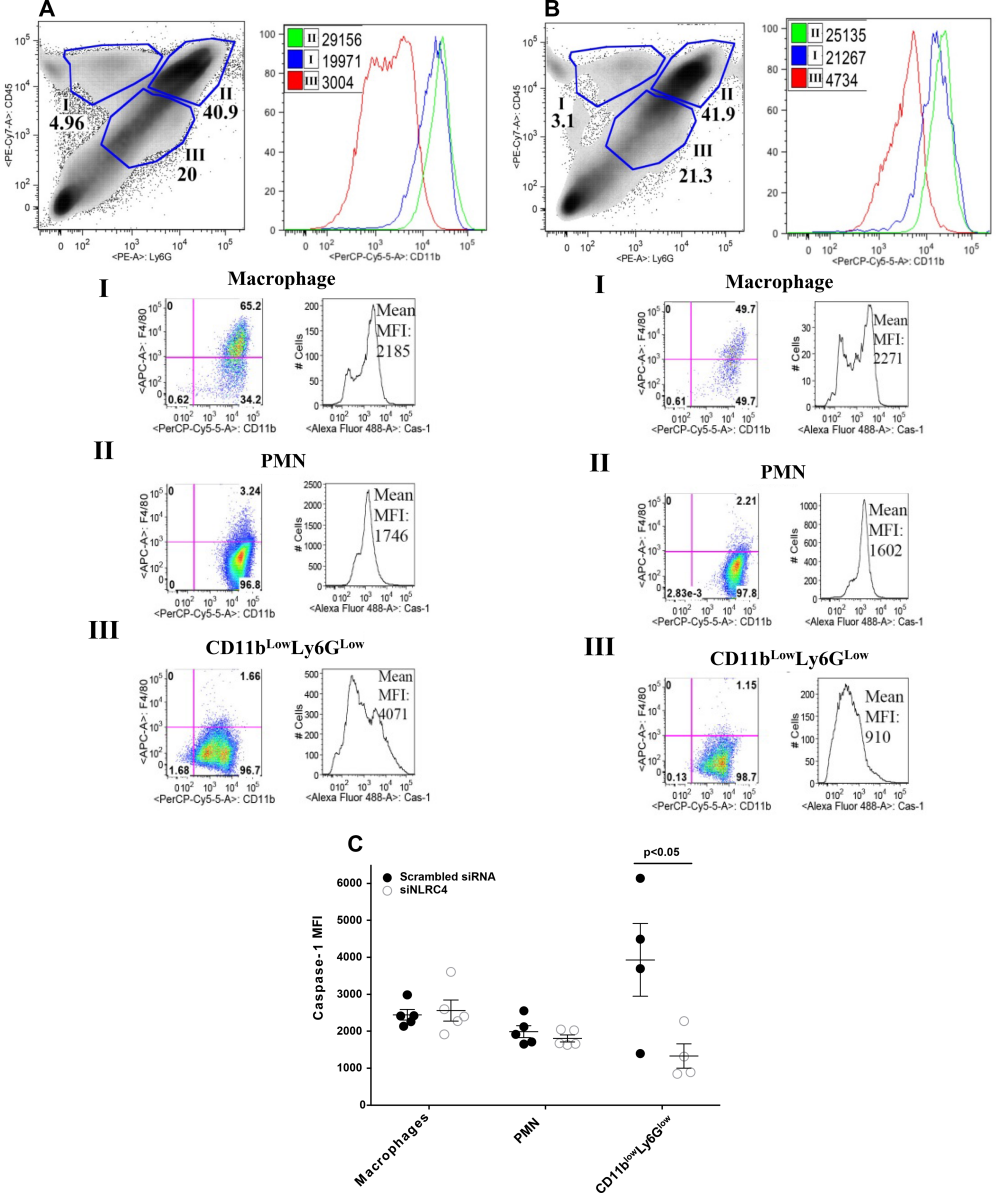
NLRC4 silencing reduces the level of activated caspase-1 in CD11b^low^Ly6G^low^ cells, but not in macrophages and PMNs in the infected corneas. (A, B) Representative density FACS plot denoting three different cell populations (I, II, III) in infected cornea from scrambled siRNA (A) and siNLRC4 (B) treated groups of mice. FAM-FLICA staining was used to detect activated caspase 1 in macrophages, PMN, and CD11b^low^Ly6G^low^ cells from the cornea of mice treated with scrambled siRNA (A) vs siNLRC4 (B) at 5 days p.i. (C) The MFI for activated caspase 1 was significantly reduced in the CD11b^low^Ly6G^low^ cells from the siNLRC4 treated mice compared to scrambled siRNA treated controls (p<0.05). Data are mean ± SEM and were analyzed using a two-tailed Student’s t test.

Since caspase-1 classically cleaves the pro-forms of IL-1beta and IL-18 to the mature active molecules, flow cytometry of cells in the infected cornea was done to investigate the effect of NLRC4 silencing on caspase-1 activation. We determined the effect of NLRC4 silencing on caspase-1 activation in CD45 expressing cells in the infected corneas at day 5 p.i. Representative density FACS plots from scrambled and siNLRC4 treated groups were gated on the basis of the expression of CD45 and Ly6G molecules. Gating strategy, including isotype controls are shown in S1 Fig. The gating strategy applied to the density FACS plots defines the selected cell populations expressing differential levels of CD45 and Ly6G molecules (Fig 3A and 3B). Our results showed that cells expressing an intermediate to low level of CD45 and Ly6G molecules (population III) also expressed lower levels of CD11b molecules in comparison to cell population I and II as determined by histogram FACS plots. We also found reduced expression of Ly6C on a Ly6G^low^ cell population, suggesting they are an immature granulocytic cell (shown in S1 Fig). Therefore, we named population III as CD11b^low^Ly6G^low^ cells. On the basis of F4/80 expression, we named population I and II as macrophages and PMN, respectively. Our results showed that macrophage and PMN populations in the infected corneas from siNLRC4 and scrambled treated groups expressed a similar MFI for caspase-1 (Fig 3C). However, a statistically significant decrease (about 50% reduction, p<0.05) in MFI for activated caspase-1 was seen in the CD11b^low^Ly6G^low^ cell population from siNLRC4 compared to scrambled treated corneas. These results suggest that NLRC4 silencing reduces caspase-1 activation in CD11b^low^Ly6G^low^ cells, but not in conventional macrophages or neutrophil populations present in the *P*. *aeruginosa* infected corneas.

### NLRC4 ELISA on sorted corneal cells

**Fig 4:**
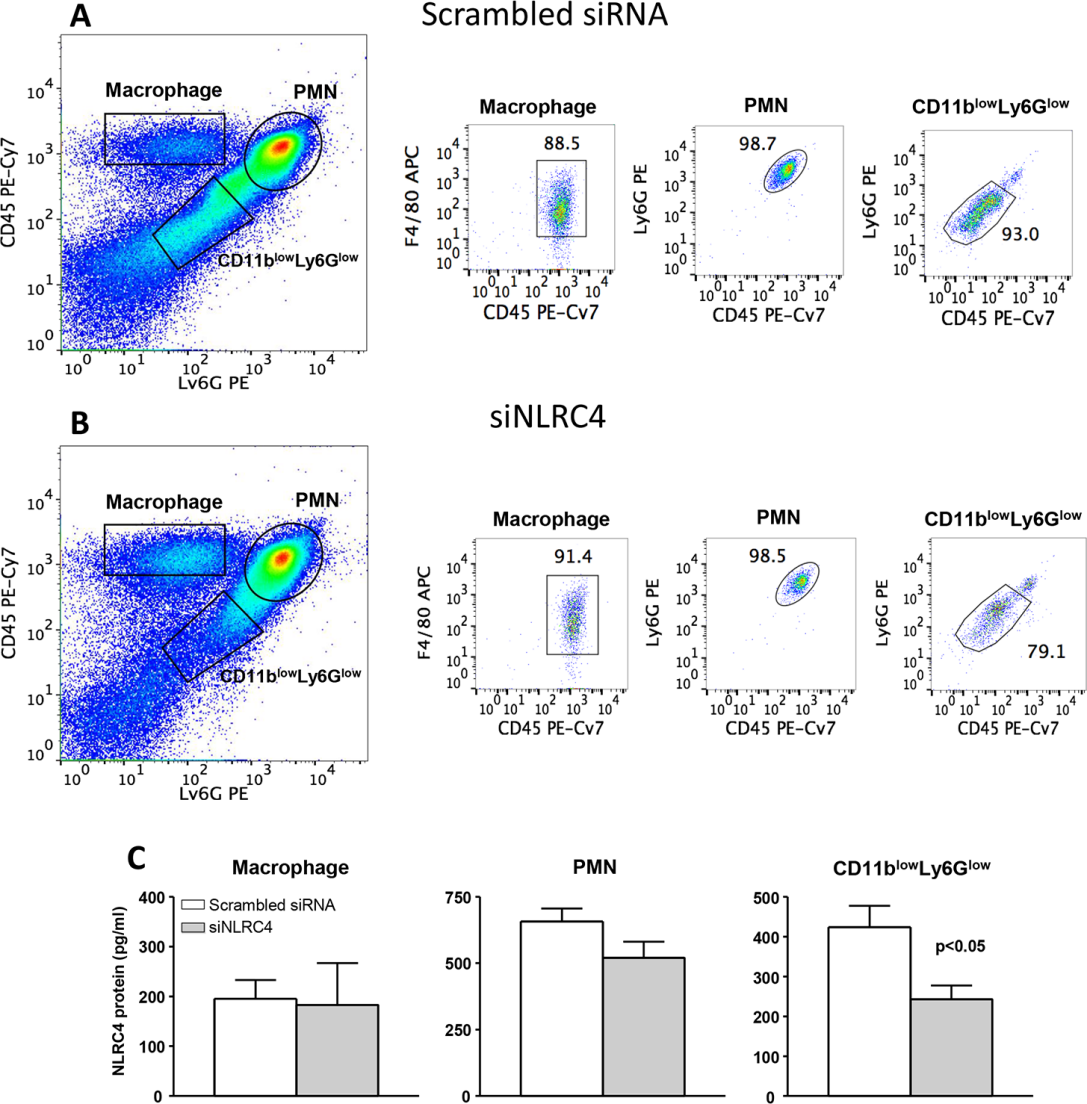
Cell sorting and NLRC4 ELISA. Flow cytometry was used to sort macrophages, PMN and CD11b^low^Ly6G^low^ cells at 5 days p.i. The purity of the cell populations are shown for cells from the corneas of scrambled siRNA (A) and siNLRC4 (B) treated mice. No significant difference in NLRC4 protein was detected in lysates from macrophages or PMN between treatment groups, however, the cell lysates from the CD11b^low^Ly6G^low^ cells sorted from the cornea of siNLRC4 treated mice had significantly less NLRC4 protein than CD11b^low^Ly6G^low^ cells from the scrambled siRNA treated mouse corneas (C) (p<0.05). ELISA data are mean ± SEM and were analyzed using a two-tailed Student’s *t* test (n = 5/group/time).

We next determined the efficacy of siNLRC4 treatment in inhibiting the amount of NLRC4 protein in macrophages, PMN and CD11b^low^Ly6G^low^ cells from the infected corneas on day 5 p.i. (Fig 4A–4C). A single cell suspension obtained from siNLRC4 and scrambled siRNA treated infected corneas was stained for the above mentioned molecules. Different cell populations were sorted on the basis of gates applied in Fig 3A and 3B. Cell lysates were prepared from an equivalent number of sorted cell populations from both groups of mice. ELISA assay was carried out to quantitate the amount of NLRC4 protein. As shown in Fig 4C, siNLRC4 treatment significantly reduced the amount of NLRC4 protein in CD11b^low^Ly6G^low^ cells (p<0.05), but not in macrophages or PMN from infected corneas.

### Detection of IL-1beta protein in cell lysates and supernatants

**Fig 5:**
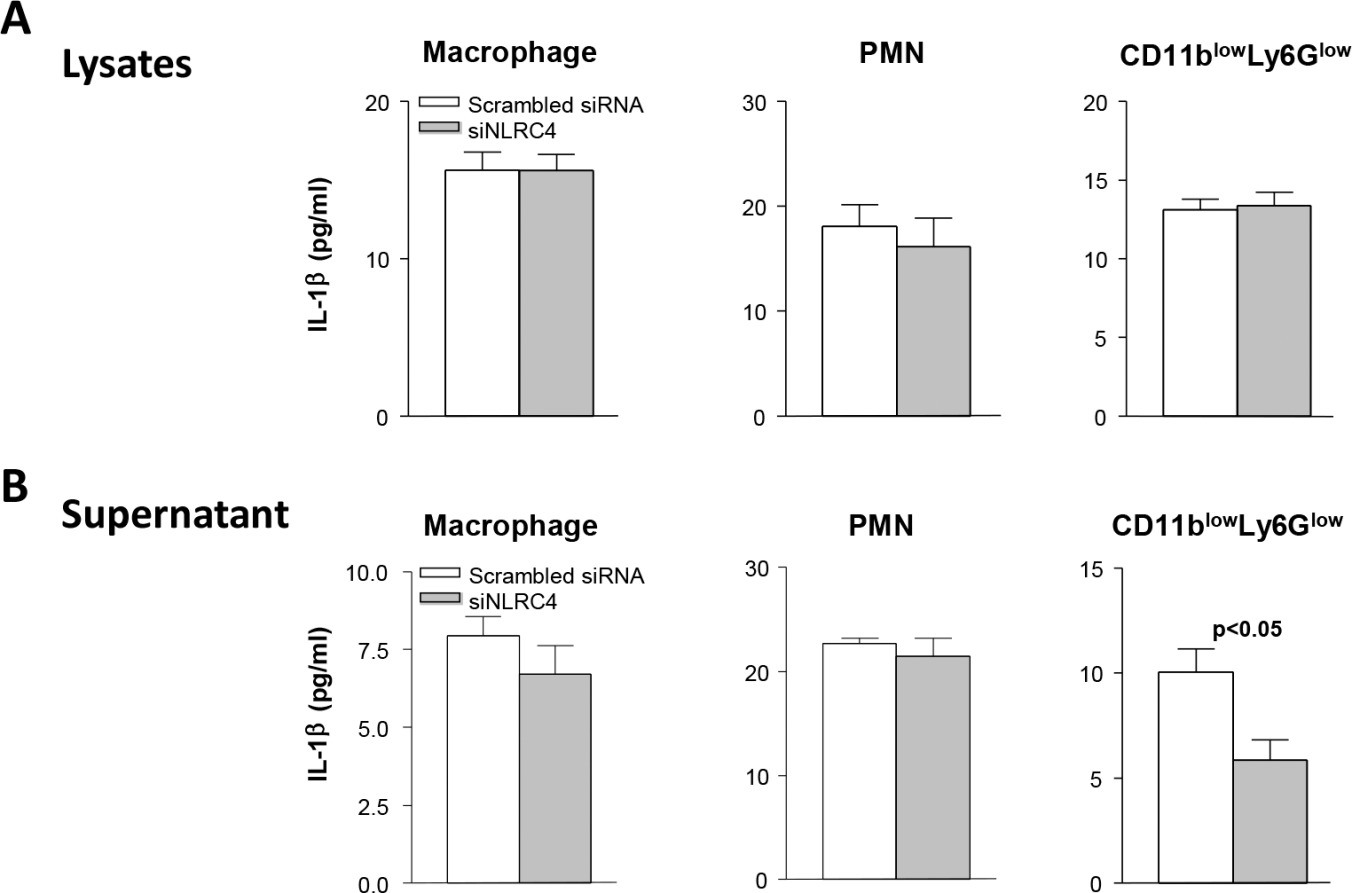
ELISA detection of IL-1beta in cell lysates and supernatants. No significant difference was seen in total IL-1beta protein in the cell lysates of macrophages, PMN, or CD11b^low^Ly6G^low^ cells sorted from siNLRC4 vs scrambled siRNA treated control mice (A). Supernatants from macrophages and PMN also showed no significant difference in total IL-1beta protein between groups. The supernatant collected from CD11b^low^Ly6G^low^ cells from siNLRC4 treated mice had significantly less total IL-1betawhen compared to the supernatant from CD11b^low^Ly6G^low^ cells from scrambled siRNA treated controls (B) (p<0.05). Data are mean ± SEM and were analyzed using a two-tailed Student’s *t* test (n = 5/group/time).

Cell lysates and supernatants were similarly analyzed by ELISA for IL-1beta and showed no difference in IL-1beta protein levels in cell lysates from macrophages, PMN or CD11b^low^ Ly6G^low^ cells sorted from scrambled siRNA and siNLRC4 groups (Fig 5A). IL-1beta ELISA assay showed no difference in IL-1beta protein levels in supernatants from macrophages or PMN between the two treatment groups. However, the supernatant collected from the CD11b^low^Ly6G^low^ cells isolated from the siNLRC4 treated mice had significantly less secreted (mature) IL-1beta protein levels compared with scrambled siRNA treated mice (p<0.05) (Fig 5B).

### Morphology and phenotype of CD11b^low^Ly6G^low^ cells

**Fig 6:**
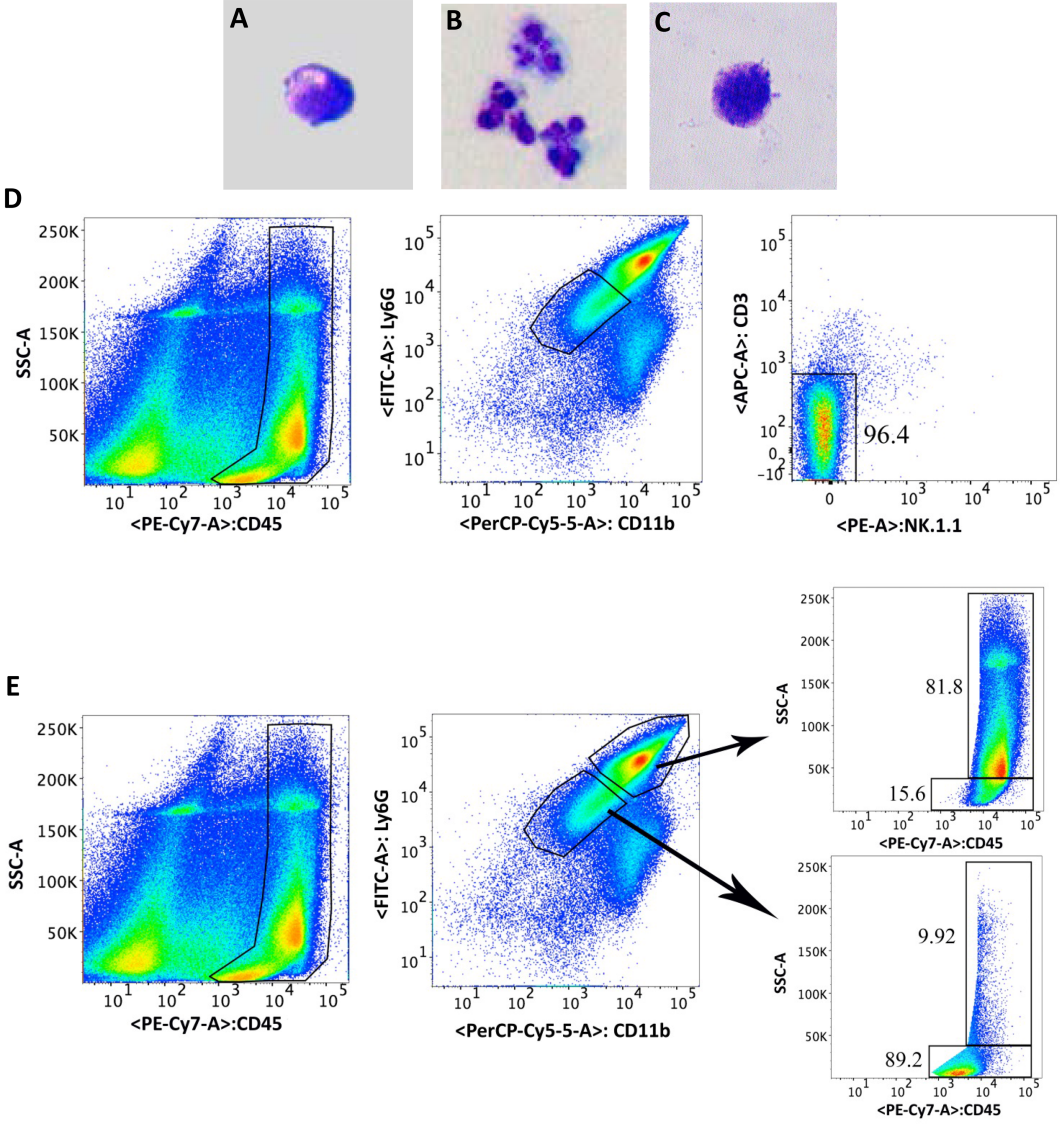
Morphology (Fig 6A-C) of the cells collected after gating for macrophages (Fig 6A), PMN (Fig 6B) and CD11b^low^Ly6G^low^ cells (Fig 6C) were consistent with expected morphology for both macrophages (Fig 6A) and PMN (Fig 6B). The CD11b^low^Ly6G^low^ cells (Fig 6C) appeared to exhibit an immature myeloid morphology. Phenotyping of the cells using FACS analysis (D) showed that the CD11b^low^Ly6G^low^ cell population did not express natural killer or T lymphocytes markers, as 96.4% of this population was negative for both CD3 and NK1.1. (E) Manual back gating applied on Ly6G^low^ and Ly6G^high^ cells showed that the majority (89.2%) of Ly6G^low^ vs 15.6% of Ly6G^high^ cells had lower side scatter, suggesting a reduced granularity of Ly6G^low^ cells.

Morphology (Fig 6A–6C) of the cells collected after gating for macrophages (Fig 6A), PMN (Fig 6B) and CD11b^low^Ly6G^low^ cells (Fig 6C) were consistent with expected morphology for both macrophages (Fig 6A) and PMN (Fig 6B). The CD11b^low^Ly6G^low^ cells (Fig 6C) exhibited a myeloid morphology. To characterize the phenotype of these CD11b^low^Ly6G^low^ cells, a single cell suspension from infected corneas on day 5 p.i. was stained for CD3 and NK1.1 cell surface markers in addition to CD45, CD11b and Ly6G molecules. FACS analysis was carried out as shown (Fig 6D and 6E). Our results showed a minimal expression of CD3 and NK1.1 molecules in CD11b^low^Ly6G^low^ cells (Fig 6D). We also noted that in comparison to CD11b^high^ Ly6G^high^ cells, the majority of CD11b^low^LY6G^low^ cells had lower side scatter, suggesting a reduced granularity of the latter cells (Fig 6E). Together, our results showed that the morphology of CD11b^low^ Ly6G^low^ cells is consistent with an immature myeloid cell phenotype.

## Discussion

*Pseudomonas aeruginosa (P*. *aeruginosa)*, is a common cause of microbial keratitis in contact lens users in developed countries [1] and is associated with ocular trauma in less industrialized countries [22, 23]. Animal models of bacterial keratitis have shed light on many of the mechanisms that the bacteria use to rapidly cause perforation of the cornea, as well as on host ocular immune defenses [2, 3]. Among murine strains used commonly, B6, a Th1 responder to many antigens [24], including *P*. *aeruginosa* [25], has been described as susceptible, because after infection, the cornea perforates within about 5 days p.i. In contrast, another inbred strain of mouse, the BALB/c, that is classed as a Th2 responder [24], when similarly infected, has a less severe disease outcome and hence have been termed resistant.

The role of NLRC4 in disease has been investigated in various models of infectious and non-infectious diseases. NLRC4 has been shown to be protective in models of respiratory melioidosis [13] and *Salmonella* infection [14]. In contrast, in a model of *Pseudomonas* induced keratitis utilizing B6 (susceptible) and NLRC4^-/-^ (on the B6 background) mice, IL-1beta processing was found to be independent of NLRC4 or caspase-1 activity for both cytotoxic and invasive strains [26].

The latter results above prompted us to focus our work on the role of the inflammasome in the resistance response of BALB/c mice to *P*. *aeruginosa* corneal infection, since outcome is not perforation in this mouse strain as it is in B6 mice. In that regard, our data provide evidence that in BALB/c mice, NLRC4 was significantly upregulated compared with the NLRP3 inflammasome after mice were infected with a cytotoxic strain (19660) of the bacteria. Since NLRC4 levels were significantly elevated compared with those of NLRP3 after infection, we focused on NLRC4. Due to the unavailability of NLRC4 knockout mice, (either commercially or privately) on the BALB/c background, and neither neutralizing antibodies or chemical inhibitors specific for NLRC4 are available, we used a silencing approach. This resulted in worsened corneal disease, a decrease in the PMN infiltrate and an increased viable bacterial plate count in the cornea of siNLRC4 vs control treated mice. Silencing NLRC4 also led to elevated levels of pro IL-1beta and reduced levels of total (mature) IL-1beta and IL-18 protein. IL-1beta has been shown to mediate PMN influx to the cornea following infection [4]. Therefore, reduction in IL-1beta in the cornea as a result of silencing NLRC4 also may lead to reduction in PMN as shown in Fig 1F accompanied by an elevated bacterial load (Fig 1G). These data agree with others who have shown that NLRC4 is required for optimal bacterial clearance in an in vivo model of *P*. *aeruginosa* lung infection [27]. Regarding IL-18, we have previously shown that in BALB/c mice, it contributes to host resistance against *P*. *aeruginosa* through induction of IFN-gamma production [28, 29] and so with diminished levels of this cytokine after silencing NLRC4, resistance (bacterial killing) would be impaired. Others also have found that BALB/c mice deficient in NLRC4 or the IL-1 receptor were highly susceptible to orogastric, but not intraperitoneal infection with Salmonella. That enhanced lethality was preceded by several factors, including but not limited to, lower neutrophil recruitment and poor intestinal pathogen clearance [30]. All of these are consistent with our findings in the keratitis model and although it is tempting to extrapolate these data in mice to the pathogenesis of human disease, one must use caution. The mouse model will require further testing, perhaps using primates whose response would either verify or dispute the conclusions reported herein.

We next determined whether PMN or macrophages were responsible for NLRC4 signaling and caspase-1 activation. Flow cytometry analysis of cells derived from the infected cornea of BALB/c mice after silencing NLRC4 was done. A population of cells (CD11b^low^Ly6G^low^) that were not PMN or macrophages was identified as important to NLRC4 signaling and caspase-1 activation. These data are in contrast to other studies which showed the macrophage as important in NLRC4 inflammasome mediated events [27, 31]. When compared with macrophages and PMN, CD11b^low^Ly6G^low^ cells had a significant reduction in caspase-1 and NLRC4 expression and produced less mature IL-1beta(and IL-18) after silencing NLRC4. They also had a minimal expression of CD3 and NK1.1 molecules, and were less granulocytic (lower side scatter) than a CD45^high^ population of cells in the infected corneas. It is well known from work in the Hendricks laboratory that the normal murine corneal stroma contains a significant number of CD45+ leukocytes [32] and that most of the cells express the CD11b marker, but not other dendritic, granulocyte, T cell or NK markers, placing them in the monocyte/macrophage lineage [32]. These data were expanded upon by the Dana lab [33] that both confirmed and extended the original work. Our studies of the infected cornea are consistent with these two reports as far as phenotypic markers, but whether these populations are similar to that which we identified in the corneal stroma after infection remains untested.

In this regard, the phenotype of the CD11b^low^Ly6G^low^ cell population is consistent with an immature myeloid cell, and more speculatively, myeloid-derived suppressor (MDSC) cells. MDSCs are a heterogeneous mixture of myeloid cells with different maturation stages classified as myeloblast, promyelocyte, and myelocytes [34]. On the basis of CD11b, Ly6G, and Ly6C expression, MDSC can broadly be categorized into granulocytic (Ly6G^+^Ly6C^low^) and monocytic (Ly6G^-^Ly6C^+^) cell subsets [35]. Our results showed reduced expression of Ly6C on a Ly6G^low^ cell population, suggesting these cells are an immature granulocytic cells (S1 Fig). NLRC4 is not expressed in Lin negative progenitor cell populations, as determined by gene expression analysis [36]. However, in our experiments, we did not gate on Lin negative cells and thus are not dealing with a pure progenitor cell population. We strongly suggest that our immature myeloid (CD11b^low^Ly6G^low^LY6C^low^) cell population is not a progenitor cell, but is at an intermediary stage before full differentiation into a granulocytic MDSC.

Mechanistically, bacterial flagellin has been shown to activate NLRC4 and TLR5 and together, maximally protected the lung mucosa against *P*. *aeruginosa*. Notably, this lung model study also reported that decreased production of biologically active caspase-1 was observed in pulmonary macrophages isolated from NLRC4 ^-/-^ and TLR5/NLRC4^-/-^but not TLR5^-/-^ mice [27]. These data in general fit well with our studies and suggest that activation of NLRC4 by *P*. *aeruginosa* (perhaps via bacterial flagellin) leads to decreased caspase-1, reduced IL-1beta and IL-18 production in CD11b^low^Ly6G^low^LY6C^low^ cells in the cornea and worsened disease.

## Supporting information

S1 Fig Gating strategy and negative controls for flow cytometry. FACS plots of *P*. *aeruginosa* infected corneal samples obtained from BALB/C mice on day 5 p.i. show the gating strategy applied for Ly6G expressing cells and the level of Ly6C expression on different cell subsets. (A) The representative FACS plot shows isotype control staining for Ly6G -PE and the gating strategy employed to distinguish Ly6G^high^ and Ly6G^low^ cell populations. (B) The histogram plot denotes MFI of buffer only caspase-1 control in the subsets of CD45 and Ly6G expressing cells, when no FAM-FLICA reagent was added to the cells. (C) The histogram plot denotes MFI of Ly6C molecules in different subsets of CD45 and Ly6G expressing cells.
(TIF)
